# Improving InterRAI care processes in long-term care homes: A constructivist grounded theory

**DOI:** 10.1177/20552076261486775

**Published:** 2026-09-04

**Authors:** Steve Iduye, Caitlin McArthur, Khaldoun Aldiabat, Jill Bally, Tracie Risling

**Affiliations:** 1 St. Francis Xavier University, RSON, Antigonish, Halifax, NS, Canada; 2 3688 Dalhousie University, School of Physiotherapy, Halifax, NS, Canada; 3 7235 University of Saskatchewan, College of Nursing, Saskatoon, Canada; 4 2129 University of Calgary, Faculty of Nursing, Calgary, AB, Canada

**Keywords:** long-term care, care improvement, care processes, quality care, residents care, InterRAI LTCF

## Abstract

**Objective:**

To provide an in-depth understanding of the care processes involving the use of the Long-Term Care Facilities (LTCF) tool in Nova Scotia, Canada. Specifically, this constructivist grounded theory study explored the perspectives of long-term care (LTC) residents, substitute decision-makers (SDMs), and nurses, with the aim of improving residents’ care.

**Methods:**

A constructivist grounded theory study was conducted in LTC homes in Nova Scotia, Canada, between September 2022 and April 2023. Data were collected through 38 semi-structured interviews with residents, SDMs, nurses, supplemented by 41 field notes and 10 analytical memos. Data were analyzed using constructivist grounded theory methods.**Results:**The emerging substantive theory was developed primarily from the perspectives of nurses and LTC management, while residents’ and SDMs’ perspectives provided contextual and supportive insights. The participants described InterRAI LTCF care process improvement through relationship-centredness, intentionality, and effective change management. If implemented properly at the point of care, these strategies could facilitate consistent care improvement for LTC residents.

**Conclusions:**

The findings have important implications for practice, policy, and future research. Care process improvement in LTC settings requires attention to relational, organizational, and technological factors. These implications are particularly relevant for LTC management, point-of-care nurses, and LTCF software designers seeking to enhance the quality and consistency of residents’ care.

Long-term care (LTC) homes provide around-the-clock care and assistance to individuals who can no longer live independently due to chronic conditions and disabilities.^1^ The residents of these LTC homes have many complex health and social needs that require specialized care.^2–5^ Therefore, meeting these care needs is crucial for enhancing their overall quality of life. According to Kodner and Kyriacou (2000),^6^ the challenges lie in navigating the maze of fragmented systems while providing comprehensive care. As such, there is a need to identify and address the gap in the care process, and this requires an in-depth understanding of the intricate relationships among care processes. In this context, Chiu et al. (2019) highlighted that understanding the complex care required by residents and the care processes is critical to enhancing their overall health outcomes.^7^ Similarly, considering each resident’s unique needs as nurses navigate the care processes is essential for achieving positive health outcomes in LTC homes.

Care processes refer to a series of interventions and activities for a specific period that involve mutual decision-making for a well-defined group of residents.^8^ Care processes within LTC are designed to enhance the well-being of LTC residents who require ongoing support and assistance due to physical, cognitive, or psychological challenges.^9^ Despite the efforts of LTC leaders, several critical problems and gaps in care processes warrant attention and resolution. A central LTC challenge is that care processes are often designed to prioritize operational efficiency over individualized care, potentially reducing residents’ autonomy and leaving them feeling disregarded and dissatisfied.^10,11^ These feelings of abandonment and neglect could worsen residents’ physical health and quality of life, highlighting the imperative of integrating comprehensive psychosocial support into care processes and minimizing fragmented care processes.^12,13^

The fragmented care processes that permeate LTC homes can disrupt the seamless coordination and continuity of care that residents require.^6^ With improper coordination and lack of continuity come different adverse health events, unnecessary hospitalizations, medical errors, medication mismanagement, delayed interventions, and prolonged recovery times for residents.^14–16^ Consequently, preventable complications may arise, exacerbating existing conditions and diminishing residents’ quality of life.^14,16^ Fragmentation of care processes can manifest in various issues, from communication gaps among healthcare professionals to disjointed handoffs during shift changes and bureaucratic hurdles that impede seamless care coordination.^16^ It can also negatively impact the emotional and psychological well-being of LTC residents, making both the residents and the family caregivers feel insecure and anxious.^9,17^ Similarly, other significant indicators of fragmented care include poor collaboration among care providers, which might be stressful for the residents and families.^18,19^ In addition, inconsistent staffing, high turnover, and inadequate training can affect the provision of necessary care and lead to fragmented care processes.^20,21^ These gaps result in suboptimal care and safety concerns.^20,22^

To optimize the fragmented care processes, the international resident assessment instrument long-term care facilities (InterRAI LTCF) tool is now widely used in many LTC homes across Canada and in several other countries. With this tool, care providers assess residents’ physical, cognitive, psychological, and social well-being and provide personalized care plans that meet their needs.^23–25^ Recent studies indicate that the tool improves care communication among healthcare professionals through standardized instruments that facilitate a collaborative approach and reduces communication breakdowns.^26–28^ In addition, using InterRAI assessment data to improve care processes supports evidence-based care planning and quality improvement by collecting and analyzing data, enabling facilities to identify trends and make changes.^26,28^ According to InterRAI Worldwide,^29^ Collaborative Action Plan (CAP), a functionality of the InterRAI LTCF tool, supports consistent, continuous care across healthcare settings. Rather than automating care planning itself, CAPs are designed to foster open conversation and shared decision-making between clinicians and the individual in LTC homes.

The InterRAI LTCF is embedded in software. In this study, the tool is referred to as LTCF, and the software (Momentum) is the digital home of the tool. Despite the tool’s undeniable benefits in LTC settings, the identified gaps in the literature challenge its potential benefits at the point of care in LTC homes. One gap concerns time constraints, potentially compromising assessment accuracy due to multitasking demands in the clinical setting by the nurses.^28,30^ Streamlining processes while maintaining quality is therefore vital to optimizing residents’ health outcomes in LTC homes. Although InterRAI assessments generate valuable data, effectively integrating and utilizing the data remains challenging for some facilities.^26,28^ In addition, the literature has not adequately answered the crucial question of how nurses and other care providers could use the tool and software to improve fragmented care processes and residents’ health outcomes. This constructivist grounded theory study, which was conducted in Nova Scotia, Canada, provides an in-depth understanding of how the tool was used in LTC homes, from the perspectives of residents, SDMs, and nurses. The study aimed to: (a) describe care processes involving the LTCF tool at the point of care; (b) identify challenges and potential improvements; and (c) develop a substantive theory that explains how to improve care provision using the LTCF tool for consistent residents’ health outcomes.

## Methods

### Design

This qualitative study employed Charmaz’s constructivist grounded theory as its research methodology and was conducted in Nova Scotia, Canada, between 2022 and 2023. Constructivist grounded theory (CGT) design enables researchers to systematically develop substantive theories rooted in empirical data.^31^ In CGT, the reality of a given phenomenon is a function of the co-construction of interpretations by participants and the researcher.^32^ Here, the researcher is not a passive observer but an active participant in the research process as the data is co-constructed with the research participants.^33,34^ In the co-construction of data, it is foremost that the researchers understand the participants’ perspectives and experiences and then constantly re-evaluate their (researchers’) interpretation of the data based on the emerging data.^34^ Rather than treating data as an objective reflection of a participant’s reality,^35^ our research team account for the social construction of data during the research process. CGT supports the idea of understanding the basic social processes in people’s complex social lives, as this is critical in theory formation.^36,37^ Since people’s social processes are inextricably linked with symbolic interactions and pragmatism, the emerging substantive theory that explains the phenomenon under study is inductive; that is, it originates from participants’ data.^37,38^ Co-creating a theory that explains LTCF process improvement in LTC homes, based on multiple subjective realities from diverse participants and extensive use of field notes and analytic memos from the researchers, makes CGT suitable for this research.

CGT was specifically selected because the purpose of this study extended beyond describing the lived experiences of the participants. The study sought to theorize the social processes by which nurses interpreted, negotiated, and responded to technological, organizational, and relational challenges as they sought to improve residents’ care. This approach enabled the analysis to move from descriptive concerns about documentation and tool usability toward a substantive theory of care process improvement grounded in participants’ actions, interactions, and meanings.

### Setting and sample

Constructivist Grounded Theory (CGT) is based on a philosophical belief that reality is not a fixed, objective truth. Instead, it is socially and historically constructed by the people involved. Within the sociocultural context of LTC homes, achieving the objectives of this study relies on the input of residents, SDMs, and nurses, who co-create subjective interpretations of the problems and potential solutions. The study took place at eight LTC homes, a for-profit organization that operates several LTC homes in Nova Scotia, Canada. The organization was an early adopter of the tool, using it for care planning with over 4,500 residents before the provincial government mandated its use in 2018. This history of early and consistent use meant the nurses in these facilities had unique institutional knowledge, making them ideal participants for the study.

Purposive sampling was the overarching method for participant inclusion in this study, and a snowball approach was used as needed to recruit participants. As the theoretical interpretation developed, theoretical sampling was employed.^37,39^ Data collection and analysis occurred simultaneously. Theoretical saturation was reached primarily within the nurse participant group, which constituted the dominant source of conceptual development. Residents and SDMs interviews provided valuable contextual and supportive insights but were not sufficient to claim independent theoretical saturation within those groups.

The participants were recruited based on the following criteria: a) Nurses who had worked directly with residents’ care for at least two years or more using the LTCF in LTC as these nurses had achieved a level of readiness with their practice within two years; b) nurses who signified interest in participating in the study by signing a consent to participate; c) LTC residents with the capacity to consent (the director of care for these LTC homes provided a list of residents who made their own health decisions and those who depended on their substitute decision makers); d) residents without the capacity to sign consent but who had SDMs in place; and, e) SDMs who held a power of attorney for LTC residents. The exclusion criteria include nurses with fewer than two years of work experience using the tool. Likewise, residents or SDMs who did not provide consent for participation were excluded from the study.

### Ethical considerations

Following the research ethics approval from the University of Saskatchewan, Canada, granted on September 06, 2022. The care directors from the participating LTC homes provided the names of interested residents and their substitute decision-makers (SDMs) for the researchers to contact. Separately, the nurses registered their interest by contacting the researchers directly as informed by the care directors. The researchers then contacted all potential participants to discuss the study, ensuring a thorough informed consent process that adhered to the University of Saskatchewan review policies. Written informed consent was obtained from all participants prior to their participation in the study. All participants were competent adults who voluntarily agreed to participate after receiving information about the study’s purpose, procedures, potential risks and benefits, and their right to withdraw at any time without consequence. Participants confidentiality and anonymity were maintained throughout the study. After addressing all questions and clarifying any concerns, the researchers obtained signed consent from those who wished to proceed with the interviews. All participants joined the study voluntarily, and no one withdrew during the interview process or after.

### Data collection and analysis

Data collection took place between September 2022 and April 2023. The researchers conducted a total of 38 interviews using a semi-structured approach with broad, open-ended questions consistent with CGT.^40^ The interviews lasted for eight months, and each participant was initially interviewed once for approximately one hour using a question guide. Only ten participants were interviewed twice during rounds of theoretical samplings. Data collection and analysis were concurrently done, which is consistent with Constructivist Grounded Theory (CGT). The data included 38 interviews, 41 field notes and 10 analytical memos. CGT is a systematic research method that uses theoretical sampling and saturation to collect data. The analysis involves an iterative process of coding, constant data comparison, and reflexivity.^40^ Coding is a foundational step that involves labelling key concepts and patterns within the data until a substantive theory emerges.^40,41^

#### Initial coding phase

During the initial coding phase, the two researchers carefully examined each phrase, sentence, and statement from participant interviews, assigning labels that reflected participants’ own words to preserve the original meaning.^37,40^ Consistent with CGT, gerunds were used to capture actions and processes. This approach helped to break down the data into manageable units and ensured alignment between the research questions and participant responses.^37,40^ As coding progressed, the researchers engaged in constant comparison, continuously comparing data segments to identify recurring patterns, and maintained memos to document emerging insights.^37,40^

#### Focused coding phase

In the focused coding stage,^37,40^ the researchers grouped related initial codes to form broader, more abstract categories. These categories were refined through continued constant comparison, allowing the researchers to identify patterns and variations within the data. Importantly, the researchers also identified and examined negative cases—instances in which participants’ views did not align with the emerging patterns.^37,40^ For example, a few participants reported positive experiences with the LTCF tool, describing the software’s interface as intuitive and supportive of care delivery. These contrasting views were attributed to participants’ prior experience with similar systems (e.g., the older RAI-MDS) and their higher digital literacy. At this stage, the researchers’ memos became more analytical, helping to track the sequence of events and recognize emerging patterns that would inform the final conceptual categories. Throughout this process, data analysis was a continuous discussion by the entire team.

#### Theoretical coding phase

In the final coding phase, the researchers moved beyond categorization to conceptualization and analysis of relationships among emerging categories and concepts, thereby constructing a theoretical framework grounded in the data.^37^ Early comparisons revealed a common theme of technical and administrative challenges in using the LTCF tool. However, some participants began to raise concerns about the actual impact of LTCF-informed care plans on residents’ health outcomes. This divergence in perspectives led the researchers to explore potential explanations. During this round of theoretical sampling, the concept of relational care emerged from the interviews. To explore this further, the researcher re-interviewed nurses who had briefly touched on the topic. After the re-interviews, the researchers reached data saturation primarily among nurse participants on this concept, as additional nurse interviews produced similar responses and no longer generated new theoretical categories. With 38 interviews, 38 fieldnotes, three aggregated fieldnotes, and ten analytic memos, the researchers determined that they had gathered sufficient information to explain the study’s aims and the emerging theory. However, residents’ and SDMs’ accounts were retained as contextual supporting data that helped illuminate care experiences and relational concerns but did not constitute an independently saturated theoretical category.

### Rigour

Credibility was ensured by aligning this study with gaps in the literature, refining the research questions through interviews, and triangulating data sources, including field notes and memos, and feedback from other qualitative research experts. Establishing originality enhanced the analysis by exploring negative cases, with theoretical sampling and reflexive memoing advancing the analysis beyond description. For resonance, participants’ words guided the coding, selected transcripts were member-checked, and preliminary findings were reviewed with LTC leadership and the research team. Usefulness was demonstrated through a transparent audit trail and findings that are both practical and theoretically relevant to similar LTC contexts.

## Findings

### Participants demographic characteristics

A total of 34 participants, representing diverse social and cultural backgrounds, were included in the study: 28 nurses, three substitute decision-makers (SDMs), and three residents. Most participants were female, comprising 27 individuals (79.4%), with the remaining seven (20.6%) being male. Most nurses worked directly on resident units as RNs and LPNs, while a few held clinical support, management, and team lead roles. Many of these nurses had extensive clinical experience and expertise with the LTCF tool and software. Regarding their experience with the tool, most nurses (67.9%) had between three and six years of experience. A second group (28.6%) had less than three years of experience, and a few (3.5%) had seven to ten years. The SDMs who participated were familiar with care expectations for their family members. Only three residents participated. Therefore, the findings and model should be interpreted primarily as reflecting nurses’ and LTC management perspectives, with resident and SDM narratives serving as contextual and supportive data.

The emerging theory is depicted as a house to emphasize that LTC is the residents’ home. The human icons in the diagram are colour-coded to differentiate the participant groups; however, the model should not be read as an equally co-constructed theory across nurses, residents, and SDMs. Rather, it represents a change process developed primarily from nurses’ and LTC management accounts, while resident and SDM perspectives provide contextual insights into care experiences and relational outcomes. The main floor of the house represents the core care process, which involves using the tool to assess health needs, planning with LTCF data, implementing LTCF care plans, and reimagining care through multiple lenses. The activities are interconnected, but the theoretical categories of intentionality and managing change were primarily derived from healthcare provider and administrator perspectives.

The quality of relationships between nurses, residents, and their SDMs was perceived as influencing care experiences and outcomes. Residents and SDMs who described positive relationships tend to report that nurses were more responsive to their needs, while those who did not experience positive relationships expressed dissatisfaction with aspects of care and its processes. These findings should be understood as relational patterns within a broader organizational context rather than as evidence that technology alone determines human relationships.

**Figure 1.**
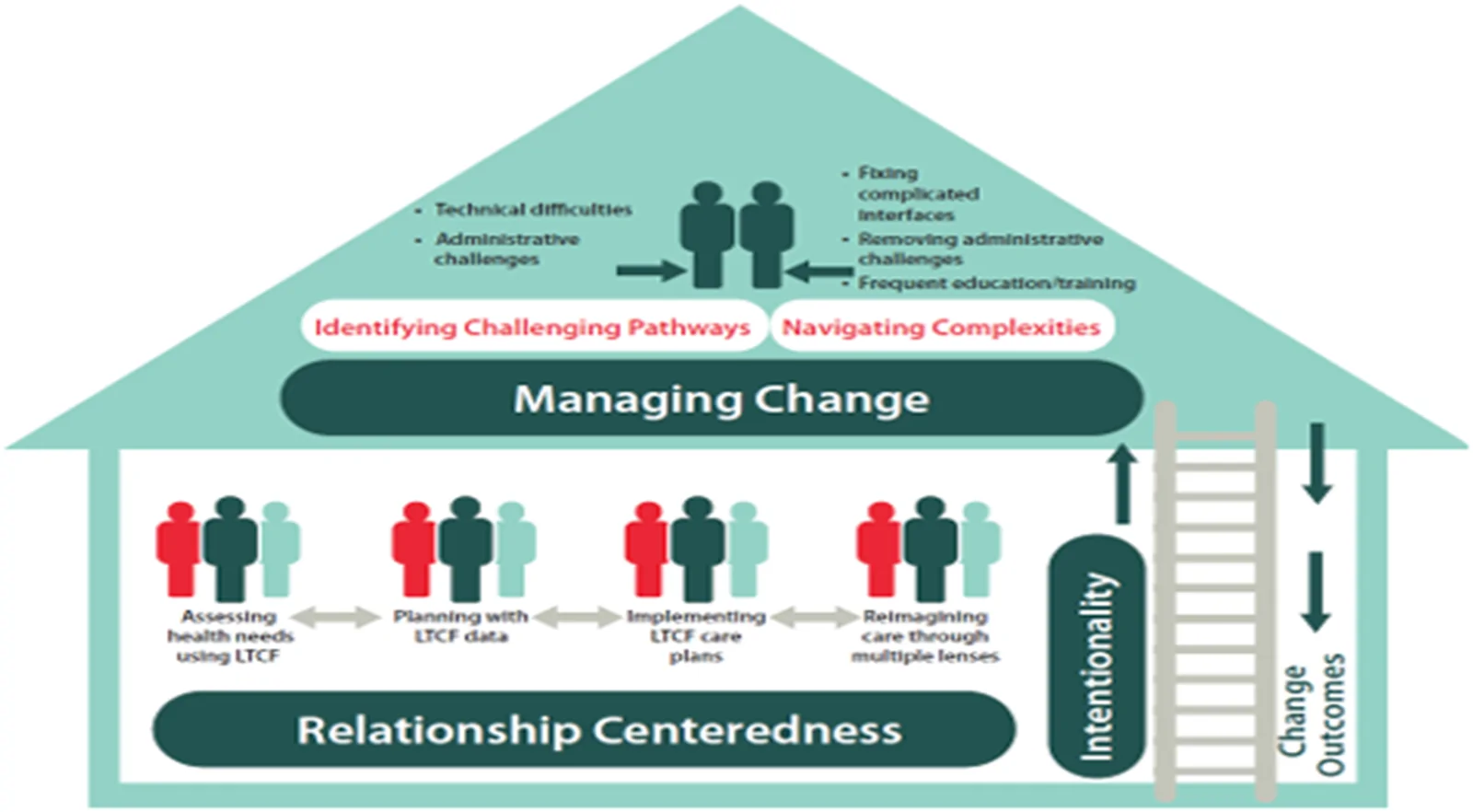
Summary of the model: Improving care process through relational caring, substantive theory.

Simply performing daily activities was not enough for nurses to meet the full needs and preferences of their residents. Therefore, an intentional shift is needed to move the care processes with an effective change management initiative. This change management process involves two equally essential steps: identifying challenging care pathways, such as technical difficulties with the LTCF software and administrative issues and proposing and executing solutions to those challenges. The way nurses and administrators manage these changes will determine the outcomes of care. These outcomes, whether positive or negative, will directly reflect in the quality of resident care and the care processes. Having summarized the model, the following sections below expatiate on the context of the findings, underlying basic social problem, and three strategies used by the participants to improve the care processes as depicted in Figure 1.

### Context of the findings: Care processes

The LTC homes’ social context situates the findings in a space shaped by the constant interactions of residents, SDMs, nurses, and the LTCF tool and software. In this LTC home environment, nurses integrated the tool and software into daily care delivery, enabling care activities to evolve, building on prior collaborative decisions and actions until they meet each resident’s needs. One of the participants described the context as follows:

So, with the [software], yeah, just what I have said, we look at the [software] on a day-to-day basis. So, we could identify if things are really being said, or have been done in the entire shift. Moreover, based on that, we could formulate what or assess what the needs of our residents in the nursing homes are, we could also plan … then go through the [software] and check on it and implement and evaluate what's happening with the residents. We do care conferences with the residents. We include the residents in our care conference, as well as the relatives, the SDMs and the entire interdisciplinary team. Then we evaluate care, looking at different views and documenting it right back in the Momentum software. So, everyone or everybody can see (RN13).

The care processes encompass ongoing, interrelated activities, but each resident’s duration of these activities varies across these phases. Care processes involve “assessing health needs using the LTCF tool, planning care based on LTCF data, implementing the LTCF care plan, and reimagining care quality from multiple perspectives”.

### Underlying basic social problem: Contention between technology and nurses

According to the participants, the basic social problem for the participants in this study was the contention between technology and nurses, a complex interaction which presented several challenges, including technical and administrative issues that impacted the use of InterRAI LTCF in resident care. Similarly, complex interfaces and unstable Wi-Fi connectivity also contributed to the technical issues. The administrative challenges stemmed from change management issues. Insufficient training, a shortage of staff, tight time frames, and restrictions to tool use were among the challenges. Consequently, this led to inconsistent care and a reduction in the use of tools for decision-making.

The care plans should be something that the nurses derive very personal stuff from. I mean, there is a space for that by adding your own thing, but they make it a little too easy to access those drop-down menus (Software) and pick whatever sounds good at the time, rather than actually picking personal things to improve the client's life (RN 1).

Ultimately, if these technical and administrative difficulties are not adequately addressed by nurses or management, they will continue to cause inefficiency and negatively affect residents’ care. The basic social process or the core category that emerged from data analysis, *Improving Care Processes through Relational Caring,* reflects the nature of participants’ social interactions and communication, emphasizing collaborative problem-solving within the care environment. To manage the main contention in the basic social process, participants used the following three relational caring strategies (categories): *Relationship-Centredness, Intentionality, and Managing Change*.

## Strategies for improving care processes

The findings are depicted in the model shown in Figure 1 above. The central challenge identified was a conflict between nurses and the LTCF software, shaped by technical difficulties, administrative barriers, and broader organizational conditions within LTC homes. To manage this concern, nurse and management participants described a core process called relational caring, which included three key subprocesses: relationship-centeredness, intentionality, and managing change. Resident and SDM accounts provided contextual evidence about how these processes were experienced at the point of care.

### Relationship-centeredness

Relationship-centredness emerged as a strategy for improving care processes in long-term care (LTC) homes. While developing therapeutic relationships with residents is a central aspect of this strategy, it also plays a critical role in fostering agency among nurses, residents and SDMs. Situating relationship-centredness in LTC practice could help to mitigate the impact of systemic and institutional challenges that compromise the quality of care. One of the participants said:

We tend to fill out these assessment tools from our own perspective. However, if we worked with a family member or clients to help fill these tools out together, we would get a much broader picture of their needs, wants, and things that we are going to aim towards (RN 9).

Prioritizing operational workflow over the resident’s engagement is not relationship-centered. In this study, the participants described characteristics such as teamwork, promoting a sense of partnership and mutuality, and a sense of consideration as relationship-centeredness. One of the SDMs mentioned that:

Well, I think, as I said to you, there's two things that really got to me. One is that the care here is family care. Moreover, that was always key to me, it's not institutionalized. Second, I get telephone calls from nurses frequently, and we're just a team working together to give him (resident) the best (SDM 1).

Participants also highlighted additional aspects of relationship-centeredness, such as embracing care practices and organizational cultures that enhance both the quality of care and quality of life for residents—an example being the concept of “family care.” Data analysis revealed inconsistencies in the delivery of such care. This meant that not all residents, or their SDMs, received the support they expected. As illustrated in the following narratives, several participants reported a lack of relationship-focused care between nurses and residents. One of the participants indicated that:

I expect consideration. I expect respect. And some of these things I don’t get … I prefer to have more Filipino and Jamaican and, and from the foreign countries, they have more care, more, more— they, they know what it’s like to— [tearful] it’s very hard here (Resident 1).

Another indication from another participant:

You rarely see a staff member come in and talk to a resident, just to come in to, you know, have a little chat. Because they are busy, and I guess their focus is where it needs to be, but … so that, more socialization, I guess, is what I would think is important (SDM 3).

These narratives implied that residents or SDMs who received care that met their needs demonstrated some levels of social connectedness with the nurses. This social connectedness is a key element in the care process. In contrast, those without these qualities did not receive care as expected. In this study, the nurse participants expressed their willingness to provide adequate support to meet residents’ needs. However, the comparison and analysis of data highlight important considerations. Residents or SDMs whose needs were met often felt a social connection with nursing staff. This sense of connectedness emerged as an important component of effective care. Conversely, those who lacked such relational elements appeared to receive care that did not fully meet their expectations. Despite the limited number of residents and SDMs, the data show that nurses and LTC administrators need to continue fostering open dialogue and creating supportive platforms that identify residents’ needs and ensure their integration into care planning and delivery. Doing this and more in LTC homes requires the second strategy that has emerged from the data, which is subprocess or category called intentionality.

### Intentionality

The second strategy, Intentionality, emerged from the nurse participants’ accounts as a key relational driver. This strategy was essential in enabling nurses to recognize and address structural challenges within the care environment that affected the quality of care provided to residents. Data analysis revealed that the LTC homes required he use of software that housed the LTCF assessment, which many nurses perceived as problematic. Addressing the complexity of these technological and administrative issues required more than technical fixes; it called for practical, grounded approaches to change management, beginning with a deliberate and reflective shift in mindset. One of the nurse manager participants reported that:

Well, for me, I think the biggest problem is how to make all the required changes to Software possible, help the staff adapt to those changes, and help the staff adapt to Software. In my view, there will be a level of commitment and intention among nurses and management staff to identify problems with the software and implement solutions (RN Manager 4).

According to nurse participants, responsibility for navigating change in long-term care (LTC) homes was shared across all staff levels. The extent to which technical issues and administrative barriers were addressed significantly influenced how effectively nurses could adopt and integrate the LTCF tool and software into their daily practice. One of the nurse manager participants said:

The hardest part for me is helping staff learn how to use Momentum's functionalities to make clinical decisions. The software is complicated for some people, but everyone needs to be ready to help identify and fix problems with the software. But if we do not all want to fix them, it is hard to give good care (RN Manager 2).

Nurse participants stressed the importance of initiating meaningful changes with purpose and intention. Leadership from LTC management will be essential for these initiatives to succeed. Nurses and frontline staff will face more challenges without managerial support. Achieving successful transformation within the LTC setting requires a unified effort, where management aligns their actions and goals to fully integrate LTCF software into daily care practices. This intentional approach to change is complex and multidimensional, involving human-computer interaction, system usability, staff training and education, relational approaches to care, technological infrastructure, and organizational governance. Therefore, the third relational caring strategy (subprocess or category) that emerged from the data focuses on managing the change.

### Managing change

The findings showed that technical difficulties involving LTCF software and administrative challenges in LTC homes significantly hindered the use and integration of the tool. Managing change involved identifying challenging pathways (problems with LTCF and software) and integrating those identified solutions (navigating complexities) into the clinical workflow and processes. One of the nurse manager participants mentioned that:

Well, for me, I think the biggest problem is how to make all the required changes in Momentum possible, and also help the staff adapt to them… everyone you talk to here knows how problematic the workflow is right now. Until there is that desire, the provision of quality care is just unattainable (RN Manager 4).

Managing change acknowledges that problems and solutions are complex, as the outcome of this stage plays a pivotal role in the successful integration of the LTCF tool and software into nurses’ daily care practices. Specifically:

It is really important to improve Momentum. If the nurses put the wrong information into the software, the care plans will be wrong too. The software can't fix the care plans on its own, which could seriously affect residents' health (RN 5 Manager).

Managing change started with identifying the challenging care pathways, which were accompanied by proactive ways of navigating complexities; so, the outcome of change management could improve care processes, if properly executed.

#### Identifying challenging care pathways

Using the LTCF tool in LTC homes has many technical and administrative challenges, primarily emanating from the software used to display the LTCF assessment and outputs. The technical challenges are particular to the software functionalities and not the actual LTCF tool. For example, one of the participants reported that:

Coming to the momentum software in such a way, care plans, if we go to the care plans, you have to type it and save, and scroll down, and again there will be interventions on the left-hand corner, you have to go, type it again, save it. So, I feel like it is really complicated. That is not step-by-step. It should be step-by-step. However, it is not the way it is. I feel like it is really complicated (RN 3).

The nurse participants reported that the most significant technical barriers were the complex interfaces that make it difficult to use the software at the point of care, as well as erratic internet access, inaccurate data, and a lack of interoperable systems. At the same time, the administrative challenges include inconsistent training and education and staffing problems. The following are two quotations from participants: “The education was probably brief. We had like one afternoon session kind of thing on it, and that was it” (RN 2). “It was peer-to-peer training. So, some of us went [and] got the formal training, and then we took it back and trained our peers. And then now it’s just through orientation and a quick overview before you start” (LPN 7).

Staffing challenges also impaired the seamless use of the LTCF tool to enhance the provision of care. As LTC homes were constantly understaffed, the participants attested to insufficient time to complete the tasks on LTCF software and experiencing overwhelming feelings of administrative burden, often resulting in inconsistent care delivery, as indicated in the responses below from three different participants:

I think there has definitely been a struggle with staffing. Trying to get these things completed when you are short-staffed is very hard. And so, I think that is a barrier that people do not have the time to be able to sit down and actually look at it comprehensively and make, you know, good assessments and good decisions about what they're going to be inputting (LPN 8).

And that's one of the bigger issues, too, because the nurses do not like using, doing the care plans on it, because they feel like they are putting information into the void. And it's never used or touched. So why am I doing this? Why am I entering this information, other than because I have to? Because it means nothing to the girls and guys on the floor that are actually doing the care (RN2).

Because of the short staff, and the changing rotations every day, there are new people on the floor. So, there is no consistency of the care and no consistency of information dissemination (SDM 3).

The staffing problems exacerbated the technical difficulties and administrative challenges in participants’ narratives. Unpacking these concerns will require a multifaceted approach to make the LTCF software use seamless LTC nurses. Having discussed the first part of the managing change strategy, let us now discuss the second part, which focuses on navigating complexities.

It is important to distinguish between general documentation burden and InterRAI/Momentum-specific usability concerns. General documentation burden refers to workload pressures common across documentation systems, including limited time, short staffing, competing clinical responsibilities, and the expectation that nurses complete assessments while maintaining direct care responsibilities. By contrast, InterRAI/Momentum-specific concerns referred to the way participants experienced this particular software interface, including cumbersome navigation, repeated saving and scrolling, reliance on drop-down menus, lack of narrative text options, limited interoperability with other documentation, and difficulty translating assessment data into usable care plans. Participants therefore did not describe documentation burden as solely caused by InterRAI; rather, they described how software-specific design issues compounded broader documentation pressures in LTC practice.

#### Navigating complexities

In this study, *“navigating complexities”* explains how participants intend to address the technical and administrative obstacles encountered using the LTCF tool and software. The nurse participants’ recommendations illustrate how the nurses sought to manage the dual challenges of intricate system interfaces and bureaucratic demands. The nurses who participated in the study recommended several modifications to the system interfaces as indicated in the following direct quotations:

“Making it easier to navigate near-misses” (LPN 4);”near misses are incidents where an error or mistake almost occurred, but the nurses intercepted them before reaching the residents; getting rid of the care plan drop-down menu” (RN 1); “adding a box for narrative text to the care plans; connecting CCAs’ documentation with LTCF software” (RN 2); and “finally, summarizing residents' care plans as a cheat sheet to save time” (LPN 8).

From the participants’ viewpoints, addressing administrative challenges involved suggestions such as increasing staffing levels for daily care and enhancing communication within care teams. One participant summarized the recommendation carefully with the following statement, “And yeah, the communication between the team members. If I have some doubts about my resident, I should not keep them in my mind. I should, I should clarify with the people, whoever knows about it. Yeah, that is what” (LPN 8).

A significant challenge in the study relates to the training required to effectively use the LTCF tool and software. Variations in computer literacy among care providers, particularly among nurses in LTC homes, present a barrier to proficiency. The findings indicate that training was insufficient for meaningful use of the tool. All nurse participants emphasized the need for comprehensive and in-depth training beyond basic data entry, as reflected in the responses below.

I feel like it would be nice to have like, even a regular kind of quarterly, even, or twice a year session with someone who knows the software really well, and can come in and say, ‘hey, guys like, this is, let us have like a roundtable session,’ so that people can ask questions about how to do things. And they can show them how to do it in real time. So better education for them, better education for the frontline staff, the RNs and LPNs who are using it every day, so they are actually comfortable using this system, and they understand they are not just collecting data, what this data is or how they can use it (RN 1).

Participants indicated that their previous training did not adequately prepare them to navigate the complexities of the LTCF software. Enhancing care quality in LTC homes—particularly regarding the effective use of this tool—will require more regular and targeted training. This training should include technical and clinical proficiency of the software and decision-making, as indicated in the following quotation:

It is essential for nurses to grasp the appropriate use of the collaborative action plans (CAPs) and scales in designing a useful care plan. In most cases, some people just dump the residents’ information into momentum with little or no understanding of how to use it to make a clinical decision or design care plans. Staff need more training in these areas, perhaps, frequent training will help (RN Manager 4).

When education effectively highlights the decision-making capabilities of the LTCF software and the LTCF tool and is provided more regularly, as nurses have suggested, it can enhance how confidently and competently nurses utilize these systems in LTC homes. Strengthening the integration of the tool and software into daily practice using these strategies could improve care processes involving the LTCF tool and the overall quality of care experienced by residents.

## Discussion

The findings of this CGT study revealed one Basic Social Process or core category, *Improving Care Process through Relational Caring*, and three relational caring strategies (categories): *Relationship-Centredness, Intentionality, and Managing Change* (as shown in Figure 1). Moving forward from the contention among the nurses and this digital assessment tool, Barnard and Sandelowski (2001)^42^ advocate for a harmonious integration of technology and relational care, highlighting that technology should be understood as both a material force and a socially constructed entity capable of enhancing patient interactions.

A key clarification arising from this analysis is that the burden of documentation should not be attributed solely to the InterRAI LTCF tool. Documentation workload is a general challenge across electronic health records, paper-based systems, and hybrid documentation environments. In this study, participants’ concerns reflected both this broader documentation burden and software-specific issues associated with the Momentum platform used to operationalize InterRAI LTCF. The distinction is important because the study does not suggest that InterRAI itself uniquely creates a documentation burden; rather, it shows how the interface, workflow design, and local implementation of the software may intensify existing workload pressures in LTC homes.

The findings should therefore be interpreted through a sociotechnical lens. The LTCF software was one factor that shaped, restricted, or guided communication and documentation practices, but it did not independently determine relationships among nurses, residents, and SDMs. Staffing shortages, rotating assignments, workload pressures, organizational culture, leadership support, and training conditions also influenced the social processes through which care was planned and delivered.

### Relational caring and relationship-centered care

The concept of relational caring as an emerging theory is interpreted in various ways across literature. To this day, no universally accepted definition of relational caring exists.^43^ However, common elements in most definitions include attentiveness to the needs of older adults and an acknowledgment of the complex, reciprocal nature of these relationships.^43^ Similarly, relational caring is often misunderstood in practice, frequently reduced to relationship-centered care that emphasizes personalized support for residents and consideration of their unique needs.^44^ Participants in this study did not explicitly define relational caring; however, their descriptions emphasized meaningful, relationship-based interactions among nurses, residents, and SDMs to uphold residents’ preferences and needs. Similarly, they pointed to the significance of dedicated nursing staff advocating for changes that could improve residents’ quality of care and health outcomes.

With these participants’ line of thought, relational caring extends beyond forming relationships among residents, nurses, and SDMs to promoting a sense of advocacy to identify and address structural issues that may affect residents’ health experiences and outcomes.^45,46^ In LTC homes, relationship-centred care provides the foundation of holistic, person-centred care that recognizes residents’ individuality and dignity. Latchem and Kitzinger (2015)^44^ noted a correlation between positive nurse-resident relationships and quality care outcomes^47^; contend that person-directed care increases residents’ engagement. Freeman et al. (2014)^48^ showed that collaborative use of InterRAI’s collaborative action plans (CAPs) enhances shared decision-making and strengthens palliative care planning. Therefore, technological tools such as LTCF should be implemented in ways that support, rather than unintentionally constrain, relationship-centered care.

### Intentionality

Intentionality in nursing practice means intentional and meaningful engagement among nurses, residents, and SDMs. Pilkington (2005)^49^ and Watson (2002)^50^ identify intentionality as a central characteristic of caring relationships within LTC settings; its application includes actions that foster empathy, presence, and human connections across systemic or technological limitations. Pilkington (2005)^49^ emphasized that intentionality must be embedded in care philosophies, especially when standardized assessments risk depersonalizing care. In this context, the LTCF tool must be employed with a clear intent to enhance, not replace nurses’ relational and clinical knowledge and skills. In this study, technical issues with LTCF software and administrative challenges frequently disrupted care delivery in LTC homes. Participants described intentionality as the deliberate effort of nurses and administrators to confront these challenges in resident care and homes. This concept reflects a collective readiness to recognize problems and act toward practical solutions, fostering significant improvements in care processes.

### Managing Change

The participants described change management in this study as addressing the technical issues, administrative challenges, and relational care implications that arise when LTC homes adopt the LTCF tool and software. The study findings indicate that changing the complicated user interfaces, including how to navigate near misses—near misses are incidents where an error or mistake almost occurred, getting rid of the care plan drop-down menu; adding a box for narrative text to the care plans; connecting CCAs’ documentation with the software; and, summarizing residents’ care plans as a cheat sheet to save time for nurses and CCAs. Previous research have shown that when new technology is added to a clinical workflow, staff become less willing and more resistant to using it.^21,51,52^ As participants indicated, identifying complex care pathways highlights the technical and managerial issues that obstruct the operational advantages of using the LTCF tool at the point of care. However, continuous staff training and education have been instrumental in mastering clinical technologies that support care provision in care settings.^53–56^ In this study, participant responses highlighted the difficulty in managing these changes, which require collaboration, commitment, and proactive problem-solving to ensure the smooth integration of this tool into daily nursing practices. In this study, it was concluded that successfully identifying and solving technical and administrative challenges within LTC homes and encouraging relational practice through change management is paramount to improving care processes overall.

The theoretical contribution of this study lies not merely in identifying isolated software problems or listing participant requests for usability improvements, but in explaining how nurse and management participants engaged in a broader process of relational caring and change management while navigating technological and organizational challenges. CGT was therefore essential because it allowed the analysis to move beyond a usability evaluation and toward a substantive theory of how care process improvement is pursued within a complex LTC environment.

Change management is challenging in LTC homes due to structural limitations and workforce shortages. There is also resistance to changing established routines. Such resistance may stem from uncertainty, perceived loss of autonomy, or concerns about job stability.^57^ Sometimes, the resistance and change itself may hinder an organization’s capacity to meet the changing environment.^58^ Successfully implementing technological tools such as the LTCF tool relies on inclusive, structured change strategies.^57,59^ To successfully implement change, strategies are needed to alleviate such concerns while facilitating an effortless transition.^60^ Relational caring and technology need not be opposing forces. Arguably, convergence is possible when change is guided by relational ethics and supported by pragmatic leadership. Change management approaches that prioritize relationship-driven values could promote collaborative workflows without jeopardizing human connections. The LTCF tool and software can improve assessments and care planning when embedded in a relational framework. To realize the tool full potential, however, change management needs to be intentional, inclusive and sensitive to context.

The participants in this study highlighted the need to reframe technological adoption as a relational, ethical, and strategic endeavour in LTC homes. Policymakers and administrators must ensure that care models balance efficiency with relational caring philosophy and that digital tools complement rather than substitute the nuanced nurse-resident relationships at the point of care in LTC homes. Care processes in LTC homes are complex and involve many interlinked events, including assessing residents’ needs, planning interventions, implementing care plans, and reimagining care quality through different lenses. Such care processes are key to improving health outcomes among LTC residents.

As indicated in the literature, this digital assessment tool can guide individualized care plans, support early identification of health concerns, and prompt timely interventions to prevent complications. However, findings of this study also suggest that the tool does not always map neatly onto the circumstances of actual care work. Nurses often described how the tool’s data can fail to capture what is happening on the floor, especially in rapidly changing or relationally complex situations. In these moments, staff must rely on relational care practices, close observation, ongoing communication, and deep knowledge of residents to compensate for gaps in the technology or to adjust care in ways that the tool cannot fully support.

To achieve better health outcomes for the residents, LTC stakeholders, government, and clinical leaders must be committed to care process and system improvement through effective change management, as the LTCF tool and software have changed the care landscape in LTC settings. Continuous staff engagement and communication, components of the change management framework, are needed to understand what is working and what is not regarding the tool’s usability at the point of care in LTC homes. The main goal of this continuous staff engagement is to elicit system and process upgrades. Repeated upgrades and elucidation of system and process requirements will help streamline workflow and improve the link between the LTCF tool, software usability, and residents’ care experience and health outcomes.

## Strengths and limitations of the study

A key strength of this study was the inclusion of participants from different roles within LTC, including residents, SDMs, LPNs, RNs, and managers. However, because the sample was heavily weighted toward nurses, the resulting substantive theory should be interpreted primarily as reflecting nurses’ and LTC management perspectives. The data set, which included 38 interviews, 41 field notes, and 10 analytical memos, supported the development of a grounded and practice-relevant explanation of how nurses and management described improvements in care processes in relation to the LTCF tool and software. The study’s primary limitation is that its findings are context-specific to the research setting, which may limit their direct application elsewhere. While the transferability of these findings is possible, those who intend to use them in another context must carefully consider the characteristics of the new setting and its participants. The low participation of residents and their SDMs, only three of each, resulted in a lack of varied perspectives from these groups. While the researcher extended recruitment to all interested residents, many sadly passed away before their scheduled interviews. Although the limited number of residents and SDMs constrained exploration of certain questions, those who participated provided rich, detailed information. Because resident and SDM data did not reach independent theoretical saturation, their accounts were used as contextual supporting data rather than as equal pillars of the emerging theory. This limitation is important when interpreting Figure 1 and the categories of intentionality and managing change, which were primarily led and described by healthcare providers and administrators.

## Implications

The research findings on technical and administrative challenges in using the LTCF tool and software have significant implications for LTC management, nurses, and software designers. The study implies that impactful change must start from the frontline staff and be supported from the top down. Improving care processes and addressing environmental factors that disrupt LTCF tool and software use are shared responsibilities of both nurses and administrators. The identified technical difficulties, in particular, highlight the need for robust, tailored training for nurses. Participants in the study recommended continuous education on care plan decision-making, specifically focusing on the software’s scales, collaborative action plans (CAPs) and care plans, and other key functionalities. Such training would provide nurses with the essential skills and knowledge to effectively use the complex clinical software and the LTCF tool. In addition, administrative challenges underscore the need to optimize workflow processes to align with software functionalities. This approach requires streamlining activities and eliminating redundant tasks to boost efficiency, productivity, and the overall quality of care. As an example, nurse participants proposed replacing multi-page care plans in the software with a “cheat sheet” that is easier to use and remember. The nurses’ narratives emphasize that support from LTC administrators and leadership is crucial for guiding and implementing such changes. Continuous improvement and innovation should be a top priority for software vendors and their LTC home clients to address evolving user needs. Ongoing collaboration between vendors and LTC homes is essential. This collaboration should include opportunities to pilot-test new features and functionalities, allowing for rapid iteration and refinement of the software based on users’ needs.

At the point-of-care level, a key policy implication is to implement policies that promote relational caring as a core culture and practice. The LTC home leadership could enact policies that provide continuous learning and allocate resources for staff training to enhance the communication and interpersonal skills of nurses, residents, and their families. On a provincial level, policymakers could integrate the principles of relational caring into broader policy frameworks. This approach would emphasize the core principles of relational caring across LTC regulations, accreditation standards, and other official policies. Future research grounded in this substantive theory should continue to refine the application and integration of the emerging theory in Nova Scotia’s LTC homes, as care processes are not static. This approach provides an opportunity for researchers, LTC administrators, frontline nurses, and LTCF software developers to collaborate. By working together, they can enhance the LTCF software’s functionality, usability, and compatibility with existing workflows across the province, ultimately contributing to better quality of care for residents.

## Conclusion

Care processes in LTC homes involve assessing health needs using the LTCF tool, planning care based on LTCF data, implementing the LTCF care plan, and reimagining quality of care from multiple perspectives. These are a series of interrelated and interdependent events, and each phase of the process should be clearly defined within the homes. Care quality improvement in LTC homes depends on relational care practices, which may be supported by relationship-centeredness, being intentional of various activities that go in and out of the care processes, as well as LTC management’s ability to continue identifying and addressing context-specific barriers and facilitators that enable the seamless use of the LTCF tool. While the LTCF tool has well-documented benefits and can influence care practices across LTC homes, it is also essential for nurses and administrators to recognize how well the tool and its software interface align with the realities of everyday care work.

## Data Availability

The data (anonymized) supporting the findings of this study are available from the corresponding author upon reasonable request.*
